# Identification of extracellular vesicle-borne periostin as a feature of muscle-invasive bladder cancer

**DOI:** 10.18632/oncotarget.8024

**Published:** 2016-03-10

**Authors:** Christopher R. Silvers, Yu-Ru Liu, Chia-Hao Wu, Hiroshi Miyamoto, Edward M. Messing, Yi-Fen Lee

**Affiliations:** ^1^ Department of Urology, University of Rochester Medical Center, Rochester NY, USA; ^2^ Departments of Pathology and Urology, Johns Hopkins University School of Medicine, Baltimore MD, USA

**Keywords:** periostin, extracellular vesicle, exosome, muscle-invasive bladder cancer and biomarker

## Abstract

Muscle-invasive bladder cancer (MIBC) is an aggressive malignancy with high mortality, and heterogeneity in MIBC results in variable clinical outcomes, posing challenges for clinical management. Extracellular vesicles (EVs) derived from MIBC have been shown to promote cancer progression. EVs derived from bladder cell lines were subjected to proteomic analysis, and periostin was chosen for further characterization due to its stage-specific gene expression profile. Knockdown of periostin by RNA interference reduces invasiveness *in vitro* and produces a rounder morphology. Importantly, treating low grade BC cells with periostin-rich EVs promotes cell aggressiveness and activates ERK oncogenic signals, and periostin suppression reverses these effects. These data suggest that MIBC might transfer periostin in an EV-mediated paracrine manner to promote the disease. To determine the potential of periostin as a bladder cancer indicator, patient urinary EVs were examined and found to have markedly higher levels of periostin than controls. In addition, immunohistochemical staining of a bladder cancer tissue microarray revealed that the presence of periostin in MIBC cells is correlated with worse prognosis. In conclusion, periostin is a component of bladder cancer cells associated with poor clinical outcome, and EVs can transfer oncogenic molecules such as periostin to affect the tumor environment and promote cancer progression.

## INTRODUCTION

Bladder cancer is the seventh most common cancer in the world, with 74,000 cases estimated to have occurred in the United States in 2014 [1]. More than 70% of newly diagnosed bladder cancers are non-muscle-invasive invasive (NMIBC), designated as stages Ta, T1, and Tis, for which the mainstay of treatment is repeated transurethral resection of bladder tumor (TURBT); however, 50–70% of tumors recur. Approximately 25–30% of bladder cancer patients have a diagnosis of muscle-invasive (MIBC) disease, and more than 50% of MIBC patients develop metastatic disease. Patients with distant metastases have only a 10% five-year survival rate [2]. The high recurrence rate and high morbidity and mortality of bladder cancer make it one of the most burdensome cancers to manage and treat [3]. It is difficult to predict the outcome of such heterogeneous tumors, and this poses challenges for clinical management; therefore, new strategies are needed to catch the disease earlier, and biomarkers for monitoring disease status will aid in the development of new treatments.

Recently, attention has turned to extracellular vesicles (EVs) as important mediators of communication among cells. Two classes of EVs secreted by cells are exosomes, produced in multivesicular endosomes and released upon endosomal fusion with the cell membrane, and microvesicles, which bud directly from the cell membrane. Vesicles of both types are membrane-bound packets bearing a select cargo of proteins, lipids, and RNA which can be delivered to specific target cells and play various roles in physiological and pathological processes [4]. There is increasing evidence that cancer-derived EVs play an integral role in cancer development and progression [5]. Numerous studies have shown that EV-mediated cargo transfer to recipient cells can affect many stages of tumor progression through communication between the tumor and the surrounding tumor microenvironment, activation of proliferative and angiogenic pathways, immune suppression, and the initiation of pre-metastatic sites [6–8]. Many molecules are not released in freely soluble form into body fluids but are encapsulated within secreted EVs. The molecular contents of EVs may reflect the releasing cell types and their status, making them useful disease biomarkers. Given the non-invasive nature of sample collection and easy accessibility of body fluids like saliva, blood, and urine, EVs promise to be excellent sources of clinical biomarkers.

Periostin (coded by the gene *POSTN*) is an extracellular matrix protein found in many normal tissues where it regulates cell adhesion and has a role in the development and maintenance of mechanical stress-bearing structures like bones, teeth, and heart valves. Periostin is overexpressed in many human cancers, and high periostin levels have been correlated with tumor proliferation, cell motility and invasion, metastasis, angiogenesis, evasion of apoptosis, clinical stage, and survival outcome [9–15]. Additionally, it has recently been proposed as a biomarker in a number of cancers [16–18]. At the molecular level, periostin activates the PI3K/Akt and/or MAP kinase pathways via interaction with integrin receptors αVβ3 and αVβ5, promoting cell adhesion, motility, and angiogenesis. However, its role in bladder cancer is less conclusive. In the current study, we identify epithelial periostin expression as a feature of bladder carcinoma associated with poor patient outcomes and characterize its oncogenic properties. In addition, our discovery of high periostin levels in bladder cancer EVs suggests its potential as a urinary biomarker of disease recurrence or progression.

## RESULTS

### Elevated periostin in muscle-invasive bladder cancer

**Figure 1:**
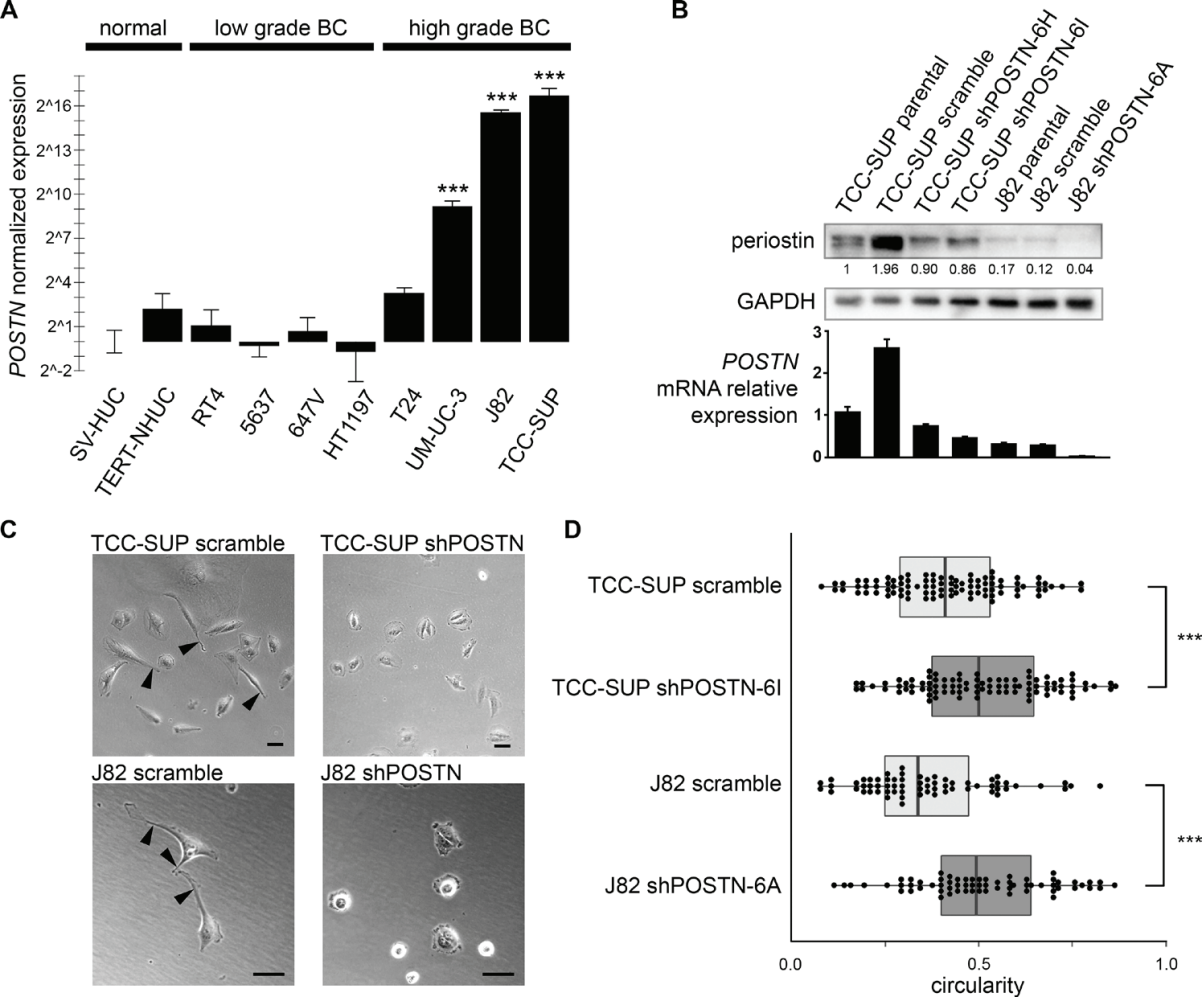
*POSTN* gene expression patterns in bladder cell lines and morphological effects of periostin suppression (**A**) Examination of *POSTN* expression in various bladder cell lines by qPCR (bars = SEM). (**B**) Periostin expression in bladder cancer cell lines TCC-SUP and J82, shPOSTN single clones, and scramble controls by Western blotting of whole cell lysates (top) and corresponding qPCR measurements of mRNA expression (bottom). Densitometry values are given for the prominent bands observed at ~81 kDa. qPCR error bars = SEM. (**C**) Phase contrast micrographs showing change in morphological phenotype in shPOSTN single clones. Membrane protrusions are indicated by arrows; bars = 50 microns. (**D**) Quantification of cell roundness in the cells in (C) using the circularity algorithm in ImageJ's particle analysis feature. Box ends correspond to the first and third quartiles.

In the screening of EV proteins by mass spectrometry detailed in our recent study [7], periostin was found to be abundant in the EVs collected from MIBC cell line TCC-SUP and was not observed in the EVs of nonmalignant urothelial cell line SV-HUC. This differential expression contradicts reports that periostin is down-regulated in invasive bladder cancer [19]. To confirm the presence of periostin in the cells as well as the EVs, we tested TCC-SUP and nine additional bladder cell lines of different stages by quantitative real-time PCR (qPCR) analysis. Periostin mRNA levels were significantly elevated in high-grade, MIBC cell lines, while lower grade, non-muscle-invasive lines had undetectable levels, as did the two immortalized normal bladder urothelium lines examined (Figure 1A). The stage-specific expression of *POSTN* mRNA in bladder cancer patient tissue samples was further examined in three published gene expression data sets aggregated by Oncomine at https://www.oncomine.org/. All data sets show that *POSTN* mRNA expression levels are significantly up-regulated in human MIBC tissue as compared to NMIBC and normal tissue [20–22] (Supplementary Figure S1).

### Periostin suppression alters bladder cancer cell morphology and reduces migration and invasion

While periostin's pro-cancer properties have been suggested in many cancers, the situation is less clear in bladder cancer. Elevated *POSTN* transcription in the high grade BC lines prompted us to examine its biological function, and we chose to knock down periostin by shRNA in the two bladder cancer cell lines in which it is most abundant, TCC-SUP and J82. Knockdown in selected single clones was confirmed by qPCR and Western blot analysis (Figure 1B).

**Figure 2:**
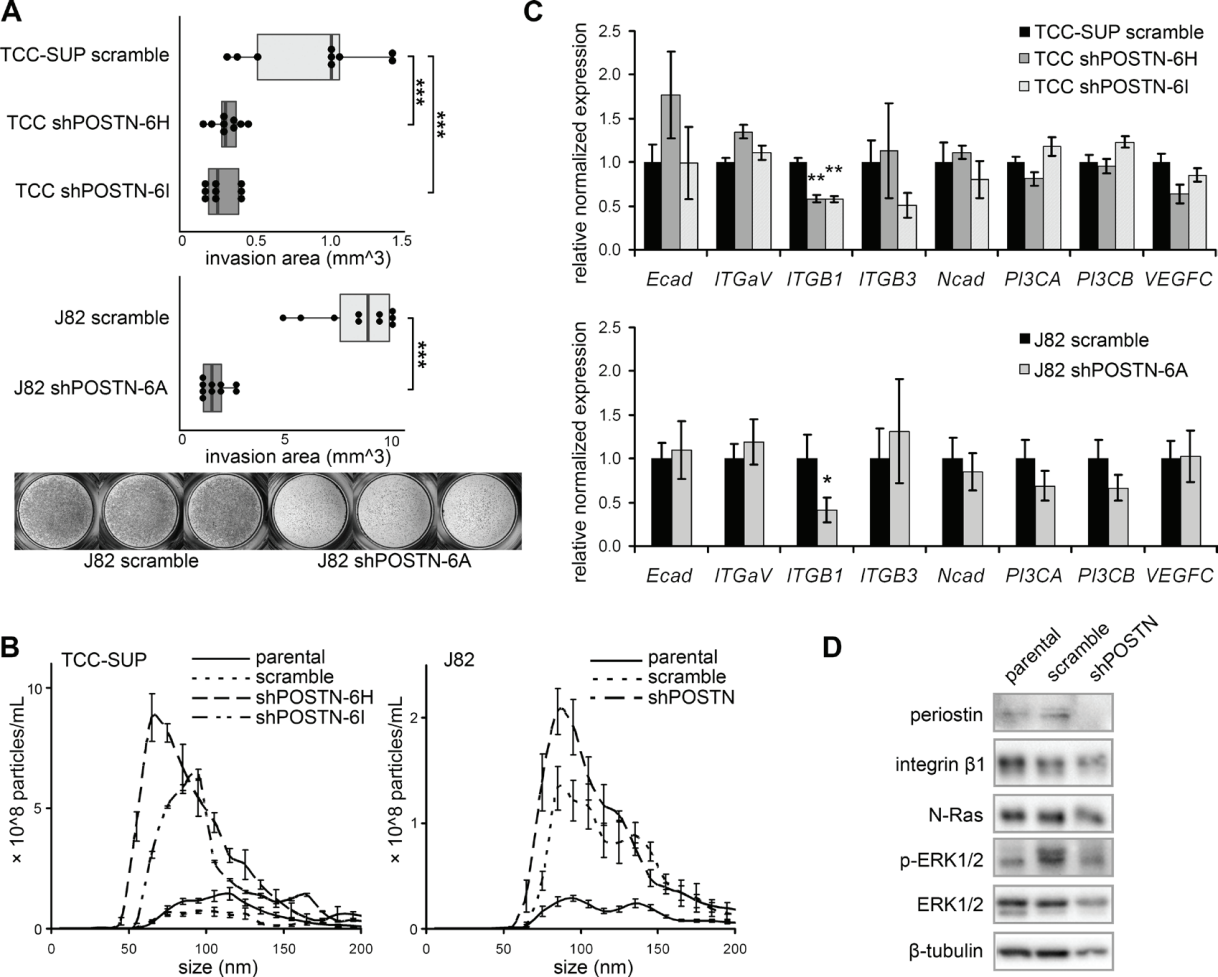
Behavioral and signaling pathway effects of periostin suppression (**A**) Behavior of shPOSTN cells in a transwell invasion assay. Quantification represents the area of toluidine blue-stained cells on the lower surfaces of the transwell inserts (illustrated in the photographs at bottom). Box ends correspond to the first and third quartiles. (**B**) NTA of size distribution and concentration of EVs isolated from shPOSTN cells and scramble controls (bars = SD). (**C**) Examination of integrin expression in TCC-SUP and J82 shPOSTN cells by qPCR (bars = SEM). (**D**) Reduction of ERK phosphorylation and N-Ras in J82 shPOSTN cells as determined by Western blot.

Periostin suppression dramatically altered cell morphology, with cells showing a loss of elongation and fewer membrane protrusions (Figure 1C, 1D). These protrusions resemble invadopodia, structures whose highly proteolytic ability to degrade extracellular matrix is thought to be critical for cancer invasion and metastasis. Indeed, we find that shPOSTN cells have markedly reduced invasion ability as compared to scramble control cells in a transwell invasion assay (Figure 2A). To our surprise, these rounded *POSTN* knockdown cells secrete more EVs than scramble J82 and TCC-SUP control cells as measured by nanoparticle tracking analysis (NTA), suggesting a possible compensation effect on EV production in response to periostin depletion (Figure 2B).

Periostin has previously been shown to stimulate cancer metastatic growth by inducing the integrin αvβ3-AKT/ERK-mediated signaling pathway. Here we find that knockdown of *POSTN* reduced integrin β1 transcription but left the rest of the integrin family unchanged (Figure 2C), suggesting that integrin β1 might be involved in periostin-mediated signaling in bladder cancer cells. Western blot analysis of *POSTN* knockdown J82 cells shows reduced N-Ras and phospho-p44/42 MAPK (ERK1/2) (Figure 2D) but no effect on activation of AKT (data not shown).

### Secretory properties of periostin

**Figure 3:**
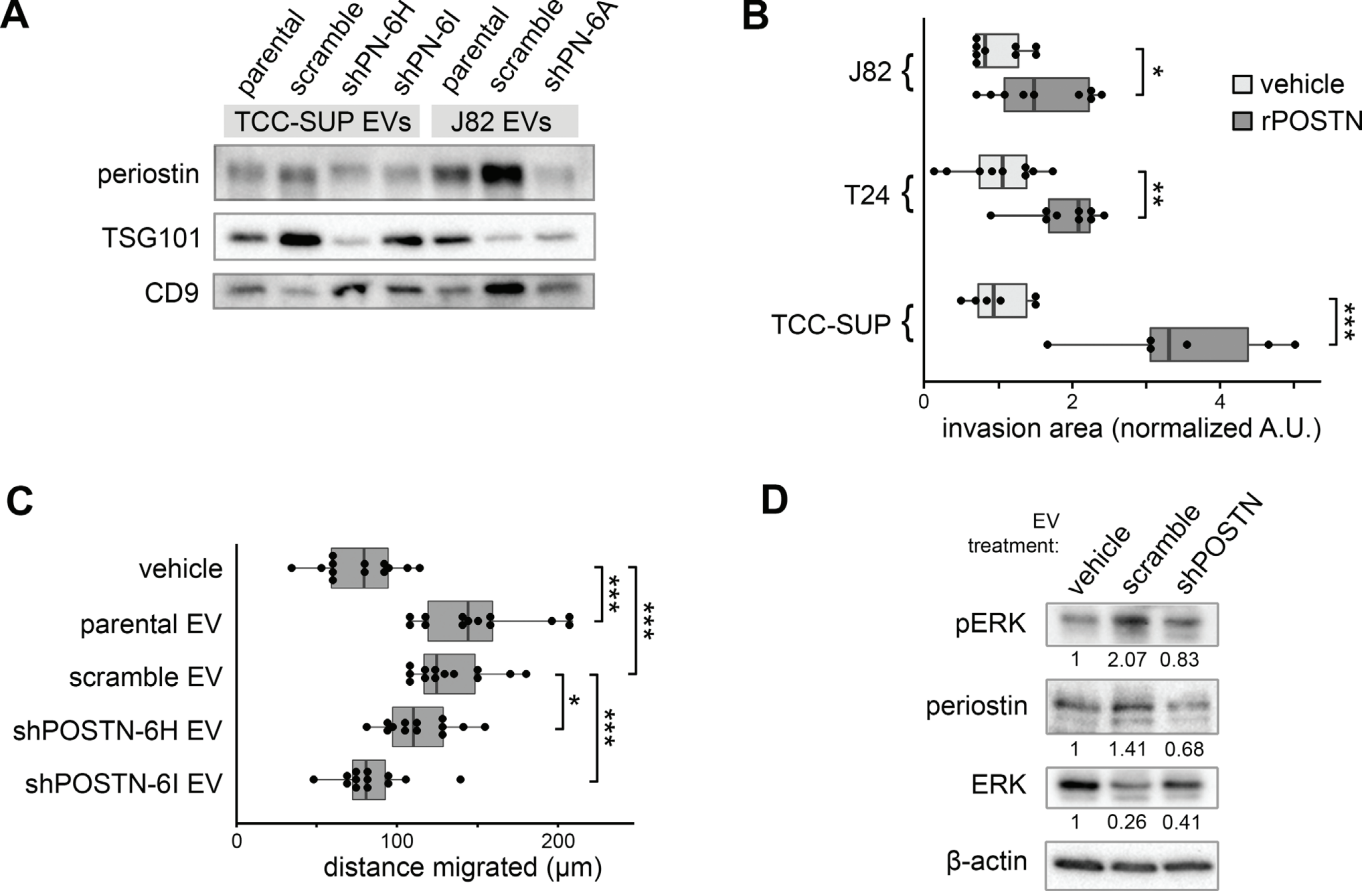
Effects of EV-borne periostin on recipient bladder cancer cells (**A**) Western blotting analysis of periostin content of EVs derived from shPOSTN cells and scramble controls. Two conventional exosome markers, TSG101 and CD9, are included. (**B**) Transwell invasion of bladder cancer cells following treatment with recombinant human periostin protein. Box ends correspond to the first and third quartiles. (**C**) Migration of 5637 cells in a scratch closure assay following incubation with higher grade TCC-SUP bladder cancer EVs illustrated in (A). (**D**) Western blot showing phosphorylation of ERK1/2 in recipient 5637 cells treated with EVs from TCC-SUP shPOSTN single clone cells or scramble cells for two hours prior to cell lysate collection.

Due to the secretory nature of periostin, it is not surprising to find it encapsulated within EVs. Prior proteomic analysis indicated that four splice variants were abundant in EVs collected from TCC-SUP cells, and Western blot analysis confirmed the presence of periostin in EVs from both TCC-SUP and J82 cells. EVs from shPOSTN cells were found to have reduced levels of periostin (Figure 3A).

In order to examine the functional significance of secretory periostin to bladder cancer, we treated three bladder cancer cell lines with human recombinant periostin protein (rPOSTN) and measured the ability of the cells to invade through basement membrane extract in a transwell invasion assay. As shown in Figure 3B, rPOSTN promoted invasion in each line. Previously, we have shown that EVs derived from TCC-SUP cells can promote migration in recipient bladder cancer cells. In order to determine whether the periostin cargo borne by EVs contributes to this effect, we treated a low-grade bladder cancer cell line, 5637, with EVs derived from TCC-SUP cells with and without *POSTN* knockdown. EVs from both parental and scramble control cells promoted 5637 cell migration, while shPOSTN EVs showed reduced promotion effects in proportion to their degree of periostin suppression (Figure 3C). Moreover, bladder cancer cells incubated with the EVs of shPOSTN bladder cancer cells contain less periostin protein after two hours than those receiving EVs from scramble control cells and exhibit less ERK phosphorylation (p-ERK), supporting the hypothesis that periostin can promote cancer progression via an EV-cargo transfer mechanism (Figure 3D).

### Periostin is abundant in MIBC urinary EVs, and epithelial periostin is associated with poor clinical outcome

**Figure 4:**
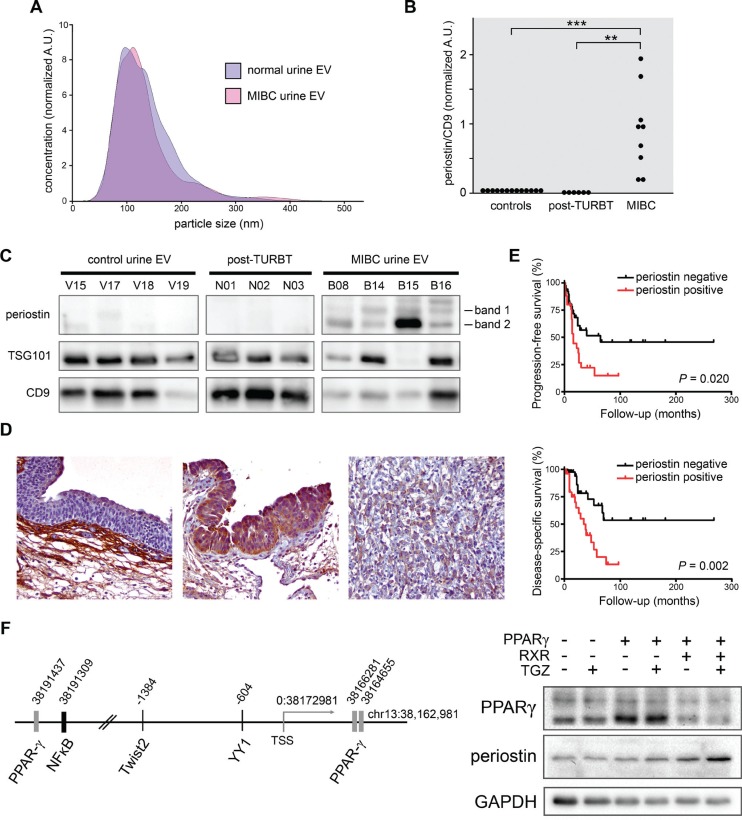
Examination of periostin in urinary EVs derived from bladder cancer patients and in MIBC tissues (**A**) NTA of EVs isolated from urine of MIBC patients and normal volunteers. Five from each group were analyzed, and one representative plot is presented. (**B**) Quantification of periostin in human urinary EVs. Densitometry of Western blot banding is presented as a sum of periostin bands 1 and 2 (at approximately 66 and 92 kDa) divided by CD9 exosome marker intensity. (**C**) Representative urinary EV samples after immunoblotting showing relatively consistent CD9 exosome marker staining and the periostin banding measured in (B). (**D**) Immunohistochemical staining of periostin protein (brown) in representative benign bladder, NMIBC, and MIBC tissues (from left to right, 200×). (**E**) Kaplan-Meier analysis of a TMA showing that positive periostin IHC staining of epithelium in MIBC tissues (scored as GIS 1-3, in red) is associated with diminished progression-free and disease-specific survival. Comparisons were made by the log-rank test. (**F**) Schematic representation of the human periostin gene locus showing putative transcription factor binding sites (left) and Western blot analysis of 293T cells transfected with pCMV-PPARγ, pCMX-RXRα, or a pCMX control vector and treated with or without troglitazone (TGZ).

The overexpression of periostin and its secretory nature make it an attractive potential body fluid biomarker for bladder cancer. To test this, we collected urine specimens from MIBC patients, NMIBC patients following TURBT, and healthy volunteers. Purified EVs were then subjected to NTA for EV quality, and periostin expression was measured in a series of Western blots. As shown in Figure 4A, EVs purified from MIBC and healthy volunteers show similar size distributions within the expected range for exosomes and microvesicles, demonstrating the ease of high quality vesicle isolation from normal and pathological specimens. Periostin immunoblotting of EVs showed undetectable or weak bands in all healthy controls and in post-TURBT NMIBC patient urine samples (Figure 4B–4C). All MIBC specimens had at least 13-fold greater periostin levels than the mean level in healthy controls; mean MIBC periostin level was 70-fold above healthy controls (Figure 4B). Additionally, urine specimens were obtained from four stage pT1 patients at the time of cystectomy. Cancers in this stage have invaded into the lamina propria but have not invaded into muscle. We found that periostin levels in EVs of pT1 cancers are greater than controls and six-fold below those of EVs from the MIBC urine specimens (*P* < 0.033). In short, the abundance of periostin in bladder cancer urinary EVs suggests its potential utility as a biomarker, and a deeper investigation of EV periostin levels in bladder cancer recurrence and stage or grade progression may be warranted.

To further test whether periostin expression levels in bladder cancer can predict patient outcome, we performed immunohistochemical staining in a tissue microarray (TMA) composed of 84 NMIBC and 64 MIBC samples. Predominant cytoplasmic staining of periostin in benign and malignant urothelial cells was observed (Figure 4D). Kaplan-Meier and log-rank tests revealed that patients with periostin present in MIBC cells had significantly higher risks for tumor progression and disease-specific mortality (Figure 4D). In addition, moderate to strong positivity (2+/3+) of periostin significantly correlated with disease progression in patients with NMIBC (Supplementary Figure S2). There was no strong correlation between periostin expression and NMIBC recurrence (Figure not shown).

### PPARγ overexpression promotes increased periostin expression

To reveal possible molecular mechanisms of periostin up-regulation in MIBC, we analyzed the periostin gene locus (−20 kb to +10 kb of chr13:38, 162, 981–38, 192, 981) using the SABiosciences text mining application and the UCSC Genome Browser (https://genome.ucsc.edu/). Among the many transcription factor binding sites predicted we found three for peroxisome proliferator-activated receptor-gamma (PPARγ), one for NFκB, and sites for other factors with cancer-associated functions (Figure 4E). Based on The Cancer Genome Atlas (TCGA) study reports on genomic abnormalities, the luminal subset of MIBCs contains features of active PPARγ signaling [23, 24]. Therefore, the activated PPARγ signal, mostly mediated through liganded PPARγ/RXR, might transcriptionally regulate the periostin gene, thus leading to the elevation found in MIBC. As expected, over-expression of PPARγ and RXR, as compared to pCMX vector control, induces periostin expression in the presence of ligand, while we found a moderate periostin induction with PPARγ alone (Figure 4F).

## DISCUSSION

In this study we provide evidence of periostin's role in the promotion of bladder cancer. Periostin up-regulation is observed in both MIBC cell lines and clinical tissue samples, periostin down-regulation via gene knockdown suppresses bladder cancer cell invasion, and treatment with rPOSTN promotes bladder cancer cell invasion. Overexpression of periostin has also been observed in a number of other human cancers. Additionally, animal studies support the pro-cancer properties of periostin *in vivo*. Kikuchi *et al.* found that gastric cancer xenografts in periostin-null mice produced smaller tumors associated with decreased ERK activation [25]. Malanchi *et al.* used *POSTN*^−/−^ mice to show that periostin expression in lung fibroblasts permits metastatic colonization by cancer stem cells by facilitating Wnt signaling [11].

Among genitourinary cancers, periostin up-regulation is associated with poor pathologic features and poor patient outcome in prostate, renal, and penile disease [19]. In contrast, periostin's role in bladder cancer has been suggested to be suppressive. Kim *et al.* reported that *POSTN* mRNA is undetectable in three selected human bladder cancer cell lines, and ectopic overexpression in SBT31A bladder cancer cells suppressed cell invasiveness and lung metastasis [26]. In our study, we screened ten human bladder cell lines, ranging from immortalized urothelial cells (SV-HUC and TERT-NHUC) and low-grade bladder cancer cell lines to MIBC cell lines, finding high *POSTN* expression in MIBC lines J82, TCC-SUP, and UMUC3, consistent with a number of cell line survey reports [27–29]. A possible explanation of this contradiction with the data from Kim *et al.* could be undefined, cell line-specific functions of different *POSTN* splice variants reported in the specific bladder cancer cell lines used [26, 30]. Moreover, the ectopic overexpression of *POSTN* in bladder cancer cells might enforce cellular environment alterations that would not occur in the normal physiological setting. Disruption of the periostin gene *in vivo* using the CRISPR/Cas system might help to clarify its roles in normal and pathological processes.

Increased EV secretion has been reported in various types of malignancy including bladder cancer [31], and many studies have revealed the roles of EVs in cell-cell communication and material transfer in immune cells and tumor cells [32–34]. Immune cells, for instance, use EVs to transfer antigens or MHC-peptide complexes to broaden the range of antigen-presenting cells and increase the efficiency of induction of immune responses [34–36]. Also, tumors utilize the EV-mediated cargo mechanism to communicate with the environment and recruit normal epithelial cells to act malignantly [37]. In our study, we find that EV production is increased in shPOSTN bladder cancer cells, while cells are rounded and less invasive (Figure 2B).

This observation suggests some possible functions of EVs and periostin. First, shPOSTN cells might compensate for the consequences of *POSTN* inhibition by activating an EV secretion pathway as a stress response. Stress related proteins such as hsp70 have been identified in EVs [38], and EVs have been shown to protect cells from oxidative stress [39]. Second, periostin has been observed in the peripheral cytoplasm of human bladder cancer cell line SBT991, as opposed to the homogeneous cytoplasm expression in mouse melanoma cell line B16F10 [30]. Rab27a functions as a vesicle docker at the peripheral cytoplasm [40]; a similar pattern of peripheral periostin expression in bladder cancer cells might also suggest a role in EV secretion.

At the molecular level, it has been reported that periostin affects cell proliferation through integrins and the PI3K/Akt pathway [41] and promotes angiogenesis through the ERK1/2-integrin αvβ3-focal adhesion kinase (FAK)-mediated signaling pathway [42]. In our study, we find that periostin knockdown cells have lower expression of integrinβ1 (Figure 2C), suggesting a positive feedback regulatory pathway between periostin and integrin β1 in bladder cancer cells. The activation of the ERK signal pathway by periostin consequently leads to bladder cancer cell invasion and migration. Interestingly, the activation of ERK by periostin/integrin β1 was also found in atrioventricular valve morphogenesis during embryonic development, possibly in a PI3K-dependent manner via FAK [43]. Targeting this periostin-mediated pathway, perhaps through inhibition of integrinβ1 and/or the ERK signal, might be beneficial for those bladder cancer patients who have elevated urinary EV periostin. A more in-depth study mapping periostin-induced cellular signaling transduction cascades, such as integrin/ERK in our data and PI3K/Akt and FAK signaling reported in other cell types, might be useful to properly develop a periostin-based therapeutic approach.

Biomarkers are increasingly employed for diagnosis, prediction, and monitoring therapy response. Attention has turned to EVs as their signature cargoes are stable within the plasma membrane and they are abundant in body fluids such as urine, blood, cerebrospinal fluid, lung epithelial fluid (accessible by bronchoalveolar lavage), saliva, and breast milk. Urinary EVs hold promise as non-invasive biomarkers to monitor disease status and treatment outcomes due to their easy accessibility and high stability compared to serum EVs. One challenge when using urinary EV proteins as bladder cancer biomarkers is determining the originating cell types of the secreted EVs. Future identification of cell-type specific EV markers is highly desirable, but in the current study, the origin of periostin in urinary EV isolates remains unclear. Healthy bladders express periostin in a belt of stroma immediately adjacent to the urothelium, and it may be that the appearance of periostin in MIBC urinary EVs is the result of tumor invasion into the stroma which allows stroma-derived periostin to access the bladder lumen. Alternatively, or in addition, it may represent up-regulation of periostin expression in the tumor cells themselves.

We find very low periostin levels in the urinary EVs after removal of tumors via TURBT, suggesting that EV periostin could be a sensitive biomarker for the presence of tumors. It would be valuable to conduct a clinical study investigating whether urinary EV periostin levels can be used to monitor tumor recurrence post TURBT. Following tumor resection, six out of six NMIBC patients had urinary EV periostin levels indistinguishable from healthy controls, and four patients retaining stage pT1 NMIBC tumors had significantly higher levels, suggesting that periostin might be a sensitive marker of tumor recurrence. Additionally, higher levels of urinary EV periostin in stages pT2-4 suggest that it might mark MIBC stage progression. In the present study, however, the low number of samples makes it impossible to draw conclusions about the specific utility of periostin as a biomarker. To evaluate this utility, increased sample sizes and long-term follow-up of clinical outcomes are needed. Additionally, there are some limitations to using urinary EVs that need further investigation. Individual urine samples vary widely in volume and concentration, and Tamm-Horsfall protein aggregation poses a hurdle to effective EV isolation and protein quantification. NTA could provide an alternative way to characterize EV size and concentration.

In summary, we have identified periostin as a feature of bladder carcinoma epithelial cells and have provided evidence that *POSTN* up-regulation may be an important driver of bladder cancer. While purification by ultracentrifuge and subsequent Western blotting are sufficient to quantify the periostin in patient urine EVs, future clinical application will benefit from more sensitive quantitative assays, such as the ELISA and Luminex assays, that require much lower sample quantity to determine the levels of EV proteins.

## MATERIALS AND METHODS

### Total RNA extraction and quantitative real-time PCR

Total RNA was collected from cells using acid guanidinium thiocyanate-phenol-chloroform extraction and quantified using spectrophotometry (NanoDrop, Thermo Scientific). First strand cDNA was synthesized using 1 μg total RNA in a 20 μL reaction using the iScript cDNA synthesis kit instructions (Bio-Rad). cDNA levels were measured in triplicate by iQ SYBR Green (Bio-Rad), and relative target expression was normalized to *GOT1* and *UBC*. Primer sequences will be available upon request.

### EV isolation

Culture supernatants and urine specimens were processed immediately after collection by serial centrifugation at 400 × *g* and 15500 × *g* to remove large debris, then stored at −80°C. EVs were isolated from thawed samples by two stages of ultracentrifugation at 200,000 × *g* for 70 minutes each, and the resulting pellets were resuspended in a small volume of DPBS. Aggregates were removed from the samples by another brief 15500 × *g* centrifugation. Final total protein concentrations of the samples were measured by Micro BCA assay (Thermo Scientific #23235), and samples were stored at −80°C.

### Nanoparticle tracking analysis

Particle size distribution and concentration in EV isolates were measured using a NanoSight NS300 (Malvern Instruments, Malvern, UK). Each sample was diluted 1:1000 in DPBS with negligible background signal and recorded into five video files of 30 seconds each. Camera level and detection threshold were kept constant among samples.

### Cell culture and cell morphology

TERT-NHUC cell line was a gift from Dr. Knowles at St James's University Hospital, and the rest of the cells were obtained from ATCC and maintained according to instructions. For *in vitro* EV collection, cell were cultured in CELLine AD 1000 bioreactor flasks (Wheaton) in EV-depleted FBS as described previously [7, 44]. For cell morphology and circularity studies, cells were seeded at low density, and photographed using a Zeiss Axio inverted microscope using a 5× objective. Fluorescence micrographs of the GFP signal (from the GIPZ lentiviral transduction) were analyzed using the particle analysis feature of ImageJ (NIH), which calculated the circularity of each cell in three distinct fields using the formula 4π × [Area]/([Perimeter]^2).

### Human subjects

Collection of urine and tissue was approved by the Institutional Review Board at the University of Rochester. Urine from patients with high-grade urothelial carcinoma of the bladder undergoing radical cystectomy was collected at the time of surgery. Additional NMIBC urine specimens were collected shortly after TURBT. Control urine specimens were provided by volunteers with no attested urological history. Written informed consent was obtained from each patient and control subject.

### RNA interference

A pool of six shRNAs against *POSTN* (Thermo Scientific) was tested, and two were selected for the best knockdown efficiency. TCC-SUP and J82 cells were transduced with shRNA pGIPZ lentiviral particles targeting *POSTN* and scrambled control particles (Thermo Scientific) following the manufacturer's instructions. Following selection in 2 μg/mL puromycin, single clones were derived by segregating single cells in 96-well plates, and expression levels of *POSTN* were confirmed by qPCR and Western blot.

### Western blots

Whole cell lysates and EV samples were separated on 12% SDS-PAGE. Sample B08 was included in each urine EV blot to allow comparison among all samples. The following antibodies were used: a polyclonal IgG against periostin (1:4000; Abcam, ab-14041); a monoclonal against TSG101 (C-2) (1:1000; Santa Cruz Biotechnology, sc-7964); a polyclonal IgG against CD9 (1:1000; System Biosciences EXOAB-CD9A-1); a monoclonal against ERK1/2 (137F5) (1:1000; Cell Signaling #4695); a monoclonal against phospho-ERK1/2 (20G11) (1:1000; Cell Signaling #4376); a polyclonal against integrin β1 (1:1000; Cell Signaling #4706); a monoclonal against N-Ras (F155) (1:200; Santa Cruz Biotechnology, sc-31); a monoclonal against PPARγ (D69) (1:1000; Cell Signaling #2430); a monoclonal against Glyceraldehyde-3-phosphate dehydrogenase (GAPDH) (6C5) (1:10K; Santa Cruz Biotechnology, sc-32233); a monoclonal against β-tubulin (9F3) (1:1000; Cell Signaling #2128); and a monoclonal against β-actin (C-4) (1:10K; Santa Cruz Biotechnology, sc-47778). Densitometry measurements were performed with Bio-Rad Image Lab software version 5.2, and the background was subtracted using a rolling ball radius of 10 mm. For periostin, two bands were measured in each lane corresponding to approximately 92 and 66 kDa. For the CD9 exosome marker, one band was measured at approximately 26 kDa. All values were normalized by reference to the B08 sample.

### Cell migration assay

5637 bladder cancer cells were grown to confluence in a 96-well plate. Uniform scratches were made in the cell layers, the wells were washed with warm culture medium, and EVs (or an equivalent volume of DPBS vehicle) were added at a concentration of 20 μg/mL in RPMI/2% FBS. Phase contrast micrographs were made using a Zeiss Axio inverted microscope immediately after EV treatment and again after four hours. Each condition had five replicates, and the experiment was performed on three occasions using unique EV samples. ImageJ software was used to measure scratch closure.

### Transwell invasion assay

Transwell polycarbonate membrane inserts with an 8 μm pore size (Corning, #3422) were coated with growth factor-reduced basement membrane extract (Trevigen Cultrex, #3433-005-02) and incubated for four hours according to the instructions. 5637 cells were serum starved for 12 hours, with or without 2 μg/mL of recombinant human periostin protein (R & D Systems, #3548-F2), and added to each insert at a concentration of 10^6^/mL. RPMI with 10% FBS was placed in the bottom well as a chemoattractant. After 16 hours, inserts were harvested, fixed in methanol, and stained with 1% toluidine blue, and photographed with a Leica MZ9.5 microscope. ImageJ was used to measure of area of toluidine-stained cells on the bottom of each insert. Each condition had three replicates, and each experiment was done three times.

### Tissue microarray

We retrieved 148 bladder specimens obtained by transurethral resection or cystectomy performed at the University of Rochester Medical Center and the Johns Hopkins Hospital. All the sections were reviewed for confirmation of original diagnoses, according to the 2004 World Health Organization/International Society of Urological Pathology classification system for urothelial neoplasms [45]. Appropriate approval from the Institutional Review Board was obtained prior to construction and use of the TMA. Bladder TMAs were constructed from formalin fixed paraffin embedded specimens as previously described [46]. These patients included 113 men and 35 women, with a mean age of 66.0 years (range: 26–89 years) at the time of surgery. The primary tumors included 11 papillary urothelial neoplasms of low malignant potential (PUNLMPs), 43 non-invasive (pTa) low-grade urothelial carcinomas, 30 non-muscle-invasive (≤ pT1) high-grade urothelial carcinomas, and 64 muscle-invasive (≥ pT2) high-grade urothelial carcinomas. Seventy-three patients, including all with muscle-invasive tumor, underwent radical cystectomy. None of the patients had received therapy with radiation or anticancer drugs pre-operatively, except for 17 cases with intravesical bacillus Calmette-Guérin treatment prior to radical cystectomy.

### Immunohistochemistry

Immunohistochemical staining was performed following deparaffinization and antigen retrieval in ~98°C citrate buffer (Vector Laboratories, H-3300). Tissues were incubated with a polyclonal antibody to periostin (1:2000 dilution, Abcam ab14041) overnight at 4°C. Staining proceeded using the standard method employing avidin-biotin complex (Vector) and the chromogen 3, 3′-Diaminobenzidine (DAB; Dako, K3466). All stains were manually scored by one pathologist (H.M.) blinded to patient identity. The German Immunoreactive Score was calculated by multiplying the percentage of immunoreactive cells (0% = 0; 1–10% = 1; 11–50% = 2; 51–80% = 3; 81–100% = 4) by staining intensity (negative = 0; weak = 1; moderate = 2; strong = 3). The immunohistochemical scores (ranging from 0–12) were considered negative (0; 0–1), weakly positive (1+; 2–4), moderately positive (2+; 6–8), and strongly positive (3+; 9–12) for periostin expression.

### Statistical analysis of TMA data

Fisher's exact test was used to evaluate the association between categorized variables. Survival rates in patients were calculated by the Kaplan-Meier method and comparison was made by log-rank test. These included comparisons of tumor recurrence in 84 patients with NMI tumor with a mean follow-up of 33.7 months (range: 3–173), tumor progression in 84 patients with NMI tumor [development of high-grade (primary PUNLMP/low-grade carcinoma only) or MI tumor] with a mean follow-up of 34.6 months (range: 3–173) and in 64 patients with MI tumor (development of local recurrence or metastatic tumor) with a mean follow-up of 35.8 months (range: 2–268), and cancer-related death in 64 patients with MI tumor with a mean follow-up of 42.8 months (range: 2–268). *P* values less than 0.05 were considered to be statistically significant.

### Transfection assay

293T cells were transfected with pCMV-PPARγ, pCMX-RXRα, or pCMX, and cells were treated with 5 nM troglitazone for 24 hours. Whole cell lysates were collected for Western blotting analysis.

## SUPPLEMENTARY MATERIALS FIGURES



## References

[1] Abdollah F, Gandaglia G, Thuret R, Schmitges J, Tian Z, Jeldres C, Passoni NM, Briganti A, Shariat SF, Perrotte P, Montorsi F, Karakiewicz PI, Sun M (2013). Incidence, survival and mortality rates of stage-specific bladder cancer in United States: a trend analysis. Cancer Epidemiol.

[2] Jemal A, Bray F, Center MM, Ferlay J, Ward E, Forman D (2011). Global cancer statistics. CA Cancer J Clin.

[3] Avritscher EB, Cooksley CD, Grossman HB, Sabichi AL, Hamblin L, Dinney CP, Elting LS (2006). Clinical model of lifetime cost of treating bladder cancer and associated complications. Urology.

[4] Raposo G, Stoorvogel W (2013). Extracellular vesicles: exosomes, microvesicles, and friends. J Cell Biol.

[5] Atay S, Godwin AK (2014). Tumor-derived exosomes: A message delivery system for tumor progression. Commun Integr Biol.

[6] Skog J, Wurdinger T, van Rijn S, Meijer DH, Gainche L, Sena-Esteves M, Curry WT, Carter BS, Krichevsky AM, Breakefield XO (2008). Glioblastoma microvesicles transport RNA and proteins that promote tumour growth and provide diagnostic biomarkers. Nat Cell Biol.

[7] Beckham CJ, Olsen J, Yin PN, Wu CH, Ting HJ, Hagen FK, Scosyrev E, Messing EM, Lee YF (2014). Bladder cancer exosomes contain EDIL-3/Del1 and facilitate cancer progression. J Urol.

[8] Tominaga N, Katsuda T, Ochiya T (2015). Micromanaging of tumor metastasis by extracellular vesicles. Semin Cell Dev Biol.

[9] Kanno A, Satoh K, Masamune A, Hirota M, Kimura K, Umino J, Hamada S, Satoh A, Egawa S, Motoi F, Unno M, Shimosegawa T (2008). Periostin, secreted from stromal cells, has biphasic effect on cell migration and correlates with the epithelial to mesenchymal transition of human pancreatic cancer cells. Int J Cancer.

[10] Bai Y, Kakudo K, Nakamura M, Ozaki T, Li Y, Liu Z, Mori I, Miyauchi A, Zhou G (2009). Loss of cellular polarity/cohesiveness in the invasive front of papillary thyroid carcinoma and periostin expression. Cancer Lett.

[11] Malanchi I, Santamaria-Martinez A, Susanto E, Peng H, Lehr HA, Delaloye JF, Huelsken J (2012). Interactions between cancer stem cells and their niche govern metastatic colonization. Nature.

[12] Ruan K, Bao S, Ouyang G (2009). The multifaceted role of periostin in tumorigenesis. Cell Mol Life Sci.

[13] Morra L, Moch H (2011). Periostin expression and epithelial-mesenchymal transition in cancer: a review and an update. Virchows Arch.

[14] Soikkeli J, Podlasz P, Yin M, Nummela P, Jahkola T, Virolainen S, Krogerus L, Heikkila P, von Smitten K, Saksela O, Holtta E (2010). Metastatic outgrowth encompasses COL-I, FN1, and POSTN up-regulation and assembly to fibrillar networks regulating cell adhesion, migration, and growth. Am J Pathol.

[15] Oskarsson T, Massague J (2012). Extracellular matrix players in metastatic niches. EMBO J.

[16] Liu Y, Shi J, Chen M, Cao YF, Liu YW, Pan J, Qi ST (2015). Periostin: a novel prognostic predictor for meningiomas. J Neurooncol.

[17] Tian B, Zhang Y, Zhang J (2014). Periostin is a new potential prognostic biomarker for glioma. Tumour Biol.

[18] Hu F, Wang W, Zhou HC, Shang XF (2014). High expression of periostin is dramatically associated with metastatic potential and poor prognosis of patients with osteosarcoma. World J Surg Oncol.

[19] Nuzzo PV, Buzzatti G, Ricci F, Rubagotti A, Argellati F, Zinoli L, Boccardo F (2014). Periostin: A Novel Prognostic and Therapeutic Target For Genitourinary Cancer?. Clin Genitourin Cancer.

[20] Blaveri E, Simko JP, Korkola JE, Brewer JL, Baehner F, Mehta K, Devries S, Koppie T, Pejavar S, Carroll P, Waldman FM (2005). Bladder cancer outcome and subtype classification by gene expression. Clin Cancer Res.

[21] Sanchez-Carbayo M, Socci ND, Lozano J, Saint F, Cordon-Cardo C (2006). Defining molecular profiles of poor outcome in patients with invasive bladder cancer using oligonucleotide microarrays. J Clin Oncol.

[22] Dyrskjot L, Kruhoffer M, Thykjaer T, Marcussen N, Jensen JL, Moller K, Orntoft TF (2004). Gene expression in the urinary bladder: a common carcinoma *in situ* gene expression signature exists disregarding histopathological classification. Cancer Res.

[23] (2014). Comprehensive molecular characterization of urothelial bladder carcinoma. Nature.

[24] Choi W, Porten S, Kim S, Willis D, Plimack ER, Hoffman-Censits J, Roth B, Cheng T, Tran M, Lee IL, Melquist J, Bondaruk J, Majewski T (2014). Identification of distinct basal and luminal subtypes of muscle-invasive bladder cancer with different sensitivities to frontline chemotherapy. Cancer cell.

[25] Kikuchi Y, Kunita A, Iwata C, Komura D, Nishiyama T, Shimazu K, Takeshita K, Shibahara J, Kii I, Morishita Y, Yashiro M, Hirakawa K, Miyazono K (2014). The niche component periostin is produced by cancer-associated fibroblasts, supporting growth of gastric cancer through ERK activation. Am J Pathol.

[26] Kim CJ, Yoshioka N, Tambe Y, Kushima R, Okada Y, Inoue H (2005). Periostin is down-regulated in high grade human bladder cancers and suppresses *in vitro* cell invasiveness and *in vivo* metastasis of cancer cells. Int J Cancer.

[27] Barretina J, Caponigro G, Stransky N, Venkatesan K, Margolin AA, Kim S, Wilson CJ, Lehar J, Kryukov GV, Sonkin D, Reddy A, Liu M, Murray L (2012). The Cancer Cell Line Encyclopedia enables predictive modelling of anticancer drug sensitivity. Nature.

[28] Garnett MJ, Edelman EJ, Heidorn SJ, Greenman CD, Dastur A, Lau KW, Greninger P, Thompson IR, Luo X, Soares J, Liu Q, Iorio F, Surdez D (2012). Systematic identification of genomic markers of drug sensitivity in cancer cells. Nature.

[29] Rothenberg SM, Mohapatra G, Rivera MN, Winokur D, Greninger P, Nitta M, Sadow PM, Sooriyakumar G, Brannigan BW, Ulman MJ, Perera RM, Wang R, Tam A (2010). A genome-wide screen for microdeletions reveals disruption of polarity complex genes in diverse human cancers. Cancer Res.

[30] Kim CJ, Isono T, Tambe Y, Chano T, Okabe H, Okada Y, Inoue H (2008). Role of alternative splicing of periostin in human bladder carcinogenesis. Int J Oncol.

[31] Franzen CA, Simms PE, Van Huis AF, Foreman KE, Kuo PC, Gupta GN (2014). Characterization of uptake and internalization of exosomes by bladder cancer cells. Biomed Res Int.

[32] Akers JC, Gonda D, Kim R, Carter BS, Chen CC (2013). Biogenesis of extracellular vesicles (EV): exosomes, microvesicles, retrovirus-like vesicles, and apoptotic bodies. J Neurooncol.

[33] Gabriel K, Ingram A, Austin R, Kapoor A, Tang D, Majeed F, Qureshi T, Al-Nedawi K (2013). Regulation of the tumor suppressor PTEN through exosomes: a diagnostic potential for prostate cancer. PloS one.

[34] Thery C, Duban L, Segura E, Veron P, Lantz O, Amigorena S (2002). Indirect activation of naive CD4+ T cells by dendritic cell-derived exosomes. Nat Immunol.

[35] Wolfers J, Lozier A, Raposo G, Regnault A, Thery C, Masurier C, Flament C, Pouzieux S, Faure F, Tursz T, Angevin E, Amigorena S, Zitvogel L (2001). Tumor-derived exosomes are a source of shared tumor rejection antigens for CTL cross-priming. Nat Med.

[36] Karlsson M, Lundin S, Dahlgren U, Kahu H, Pettersson I, Telemo E (2001). “Tolerosomes” are produced by intestinal epithelial cells. Eur J Immunol.

[37] Roma-Rodrigues C, Fernandes AR, Baptista PV (2014). Exosome in tumour microenvironment: overview of the crosstalk between normal and cancer cells. Biomed Res Int.

[38] Thery C, Regnault A, Garin J, Wolfers J, Zitvogel L, Ricciardi-Castagnoli P, Raposo G, Amigorena S (1999). Molecular characterization of dendritic cell-derived exosomes. Selective accumulation of the heat shock protein hsc73. J Cell Biol.

[39] Eldh M, Ekstrom K, Valadi H, Sjostrand M, Olsson B, Jernas M, Lotvall J (2010). Exosomes communicate protective messages during oxidative stress; possible role of exosomal shuttle RNA. PloS one.

[40] Ostrowski M, Carmo NB, Krumeich S, Fanget I, Raposo G, Savina A, Moita CF, Schauer K, Hume AN, Freitas RP, Goud B, Benaroch P, Hacohen N (2010). Rab27a and Rab27b control different steps of the exosome secretion pathway. Nat Cell Biol.

[41] Yang L, Serada S, Fujimoto M, Terao M, Kotobuki Y, Kitaba S, Matsui S, Kudo A, Naka T, Murota H, Katayama I (2012). Periostin facilitates skin sclerosis via PI3K/Akt dependent mechanism in a mouse model of scleroderma. PloS one.

[42] Watanabe T, Yasue A, Fujihara S, Tanaka E (2012). PERIOSTIN regulates MMP-2 expression via the alphavbeta3 integrin/ERK pathway in human periodontal ligament cells. Arch Oral Biol.

[43] Ghatak S, Misra S, Norris RA, Moreno-Rodriguez RA, Hoffman S, Levine RA, Hascall VC, Markwald RR (2014). Periostin induces intracellular cross-talk between kinases and hyaluronan in atrioventricular valvulogenesis. J Biol Chem.

[44] Mitchell JP, Court J, Mason MD, Tabi Z, Clayton A (2008). Increased exosome production from tumour cell cultures using the Integra CELLine Culture System. J Immunol Methods.

[45] Miyamoto H, Miller JS, Fajardo DA, Lee TK, Netto GJ, Epstein JI (2010). Non-invasive papillary urothelial neoplasms: the 2004 WHO/ISUP classification system. Pathol Int.

[46] Miyamoto H, Yao JL, Chaux A, Zheng Y, Hsu I, Izumi K, Chang C, Messing EM, Netto GJ, Yeh S (2012). Expression of androgen and oestrogen receptors and its prognostic significance in urothelial neoplasm of the urinary bladder. BJU Int.

