# Supplementary material for: Identification of extracellular vesicle-borne periostin as a feature of muscle-invasive bladder cancer
Source: Oncotarget. 2016 Mar 10;7(17):23335–45. doi: 10.18632/oncotarget.8024 (PMC5029630; doi:10.18632/oncotarget.8024)
Supplement: Supplementary file 1 [file oncotarget-07-23335-s001.pdf]

Identification of extracellular vesicle-borne periostin as a feature of muscle invasive bladder cancer

Supplementary Materials

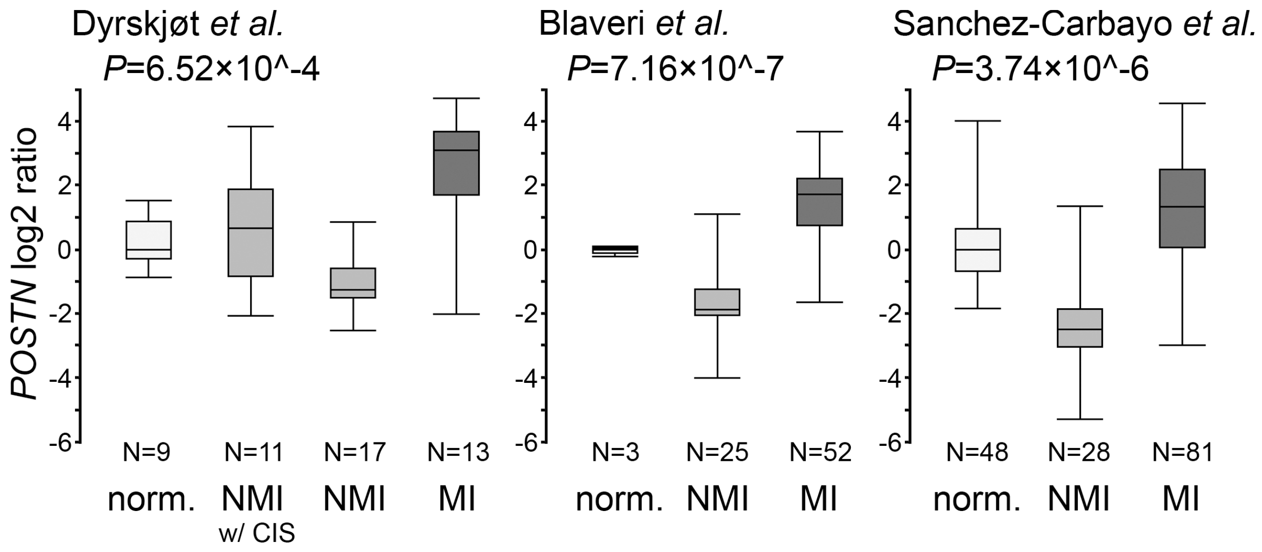

Supplementary Figure S1: *POSTN* expression levels in tissue biopsies as reported in three independent datasets. Datasets were examined using the Oncomine microarray database and data-mining platform. Box ends correspond to the first and third quartiles. *P* values are given for MIBC vs. normal.

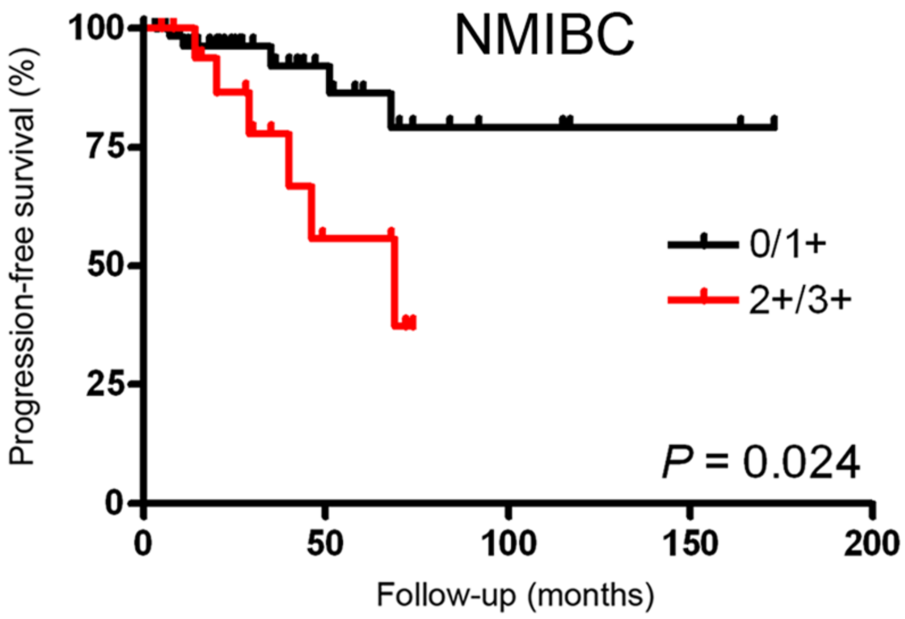

Supplementary Figure S2: Kaplan-meier analysis of progression-free survival in patients with NMIBC compared to levels of periostin expression. Comparisons were made by the log-rank test.
